# Positive Welfare and the Like: Distinct Views and a Proposed Framework

**DOI:** 10.3389/fvets.2020.00370

**Published:** 2020-07-02

**Authors:** Jean-Loup Rault, Sara Hintze, Irene Camerlink, Jason Richard Yee

**Affiliations:** ^1^Institute of Animal Welfare Science, University of Veterinary Medicine, Vienna, Austria; ^2^Division of Livestock Sciences, Department of Sustainable Agricultural Systems, University of Natural Resources and Life Sciences, Vienna, Austria

**Keywords:** animal welfare, good life, happiness, life worth living, quality of life, reward, well-being

## Abstract

Positive welfare and related terms such as good welfare, happiness, and a good life are increasingly used in the animal welfare science literature. Overall, they highlight the welfare benefits of providing animals opportunities for positive experiences, beyond the alleviation of suffering. However, the various terms remain loosely defined and are sometimes used interchangeably, resulting in discrepancy. In this perspective article, we lay out the terms and concepts used in the literature. We identify two distinct views: “*hedonic positive welfare*,” arising from likes and wants and their positive outcomes on welfare; and “*positive welfare balance*,” as an overall positive welfare state based on positive experiences outweighing negative ones. *Eudaimonia*, satisfaction with one's life, may emerge as a third view. We propose a framework that is applicable across the different views. The “Vienna Framework” outlines different facets: frequency, duration, arousal, context, previous experience, individual differences, sense of agency, and long-term benefit. The framework aims to encourage researchers to consider the relevance of these facets for their own research, to indicate how the facets are affected by different interventions (e.g., greater sense of agency in enriched compared to non-enriched animals), or to compare different topics with respect to the different facets (e.g., high arousal of play behavior and low arousal of social affiliation). We encourage researchers to carefully consider and clearly state how their work falls along these views and facets, conceptually, and operationally. This should prevent dilution of the meaning of positive welfare and thereby preserve its potential to improve the welfare of animals.

## Introduction

Animal welfare science was born of the need to address societal concerns (1). The field has primarily focused for the last 50 years on negative or detrimental aspects to animal welfare, specifically eliminating or minimizing pain, fear, distress, and other forms of suffering (1, 2), in line with Bentham's (3) “Can they suffer?” question. In contrast, positive welfare and related concepts have received increasing attention recently, mostly since the start of the millennium when several researchers started to address the topic in rapid succession [e.g., (2, 4–6)]. Positive aspects of welfare have nevertheless formed part of many definitions of animal welfare (7–10). In fact, Bentham (11) also mentioned that each human aims to maximize her/his happiness or pleasure and minimize her/his suffering, and we postulate that he would have argued similarly for animals.

In this perspective article, we see positive welfare as a concept that fits under animal welfare and that focuses on a specific and overlooked part of it. The rationale for animal welfare improvement is not just based on what the animal suffers from or lacks, but also on the welfare benefits of providing opportunities for positive experiences (2). Deprivation of certain opportunities might not necessarily cause suffering but it withholds the potential for positive welfare.

Positive welfare is sometimes perceived as luxury or accessory to the fulfillment of more basic needs such as safety and food. However, the various needs do not strictly build on each other as initially proposed by Maslow's *Hierarchy of needs* (12). Rather, in humans, the various needs have relatively independent effects on subjective well-being (13). For example, “a person can gain well-being by meeting psychosocial needs regardless of whether his or her basic needs are fully met” (13). Therefore, positive welfare cannot simply be considered the opposite of suffering because they involve qualitatively different constructs.

Positive welfare fits with how the public conceptualizes animal welfare. Lay people generally associate animal welfare with the provision of opportunities for positive experiences with an *a priori* assumption that one should not inflict pain or suffering on an animal (14, 15). This contrasts with the bulk of the scientific research on animal welfare, which is to date largely concerned with the alleviation of suffering. This discrepancy may also be exacerbated by the negative lexical bias in animal welfare science predominantly using terms such as “lack,” “prevention,”, or “freedom from” instead of “provision,”, “fulfillment” and “freedom to”; although lexical bias toward negative states is common in many languages (16).

The way one conceptualizes and studies positive welfare is inherently linked to one's own ethical views, i.e., value-based judgements, as is the case with other animal welfare aspects (1, 17). For instance, lay people and experts systematically disagreed on what a good animal life is (18), as lay people referred to a broader concept of animal welfare encompassing “living a natural life,” while experts focused on the animals' subjective experiences. This finding has been supported by other studies (15, 19) and links to the different ethical concerns regarding animal welfare (10), namely “biological functioning,” “affective states” and “natural behavior.” Consequently, it is not surprising that there are multiple co-existing concepts and definitions of positive welfare (as for animal welfare in general) that are influenced by ethical views from different interest groups including the scientific community, animal users, and society (18). Nevertheless, discriminating between the many uses of the term “positive welfare” is crucial for science to make meaningful contributions (17).

This perspective article aims to set some guidelines to help clarify the field of positive welfare. Toward this aim, we lay out the terms and concepts used in the scientific literature. Despite the heterogeneity in the literature, we identify distinct views on positive welfare to realize how one understands positive welfare. We propose a framework to map one's own operational position on the positive welfare research landscape, helping to lay out differences and state how one studies positive welfare.

## Terminology

Positive welfare is often written between quotation marks, “positive welfare”, symbolizing the sensitivity around the scientific use of this term. A variety of terms have been used to refer to positive welfare and related concepts (Table 1). These include positive welfare itself (2, 15, 20–24), good welfare (7, 9), happiness (21, 25), quality of life (5, 26), a good life (5, 18, 27), a life worth living (5, 28, 29), and various combinations such as “positive aspects of animal welfare” (*Animals* journal Special Issue 2019). Conceptual and operational definitions of these different terms are rarely given and there is an expanding number of interchangeably used terms. Lawrence et al. (30) in a recent review of the literature also found that very few papers on positive welfare developed the concept or provided a definition, with “significant overlap between the concepts and ideas that have variously contributed to positive animal welfare.” The current literature proved heterogeneous but we found implicit similarities as illustrated in Table 1 and discussed in the next section.

**Table 1:** Examples of terms and concepts related to positive welfare.

**Term**	**Definition**	**Approach**
Positive emotions	“Emotions associated with positively reinforcing situations” (8)	“Autonomic emotional responses do not define welfare in themselves […]. They are only useful in the assessment of an animal's emotional state if they can be shown to be reliably linked to situations that animals find negatively [or positively] reinforcing.” (8) “Positive emotions can be separated into three temporal categories: (i) past (e.g., post-consummatory satisfaction), (ii) present (e.g., pleasant sensory activity), and (iii) future (e.g., positive expectation, anticipatory joy)” (4)
Positive affective states	“States that are experienced as pleasant” (31)	Linking (on a two-dimensional scale) negative affect to survival situations (what the animal needs) and positive affect to opportunity situations (what the animal wants/likes) (31)
Animal pleasure	Indirectly stated: good feelings (32)	Observable by (rewarding) behavior, like playing, eating, mating and touching (32)
Happiness	“How animals feel most of the time, i.e., the balance of positive and negative affect” (25)	“Happiness is a long-term, typically stable state, which reflects how one feels most of the time, that is, the typical level of affect.” It is measurable by behavior, vocalizations and physiological correlates (25)
Good welfare	“An animal experiences good welfare if the animal is healthy, comfortable, well-nourished, safe, is not suffering from unpleasant states such as pain, fear and distress, and is able to express behaviors that are important for its physical and mental state” (7) “Healthy animals that have what they want” (8)	“Good animal welfare requires disease prevention and appropriate veterinary care, shelter, management and nutrition, a stimulating and safe environment, humane handling and humane slaughter or killing” (7) Good welfare can be based on the answers to two questions: Q1: Will it improve animal health? and Q2: Will it give the animals something they want? (8) “i.e., that the primary needs of the animals are met.” “Good welfare is not simply the absence of negative experiences, but rather is primarily the presence of positive experiences such as pleasure.” Positive experiences are a core component of good welfare; and is expressed in motivation to play (4) Good welfare can be measured by behavioral or physiological indicators of pleasure and preference testing: (9)
Positive welfare	What the animal likes (positive affective state) and what the animal wants (positive motivation to obtain a resource) (2) “Mental and physical states that exceed what is necessary for immediate survival” (24) “Positive animal welfare is a relatively new concept which promotes the welfare benefits of providing animals with greater opportunities for positive experiences, in addition to minimizing negative experiences” (33)	Positive welfare should be evaluated on the basis of input (physical resources that are required or valued by an animal) as well as output (positive outcomes such as behavioral responses, cognitive processes and physiological markers) (2) Positive welfare relates to motivation, emotion, agency, exploration, learning, play behavior (24)
Quality of life (QoL)	“Quality of life is a multidimensional, experiential continuum. It comprises an array of affective states, broadly classifiable as comfort-discomfort and pleasure states. […] Quality of life is a uniquely individual experience and should be measured from the perspective of the individual” (26) “Quality of life is the subjective and dynamic evaluation by the individual of its circumstances (internal and external) and the extent to which these meet its expectations (that may be innate or learned and that may or may not include anticipation of future events), which results in, or includes, an affective (emotional) response to those circumstances (the evaluation may be a conscious or an unconscious process, with a complexity appropriate to the cognitive capacity of the individual)” (34)	QoL includes subjective experience, affect, and nature of the individual experiences. Concepts: (dis)comfort, needs, control, social relationships, health, and stress. McMillan proposes that in animals QoL is solely based on affect, and measured on a 2-dimensional scale of comfort-discomfort and pleasure. (26) “QoL is complex and subjective and can only properly be measured from the individual's perspective.” QoL has been measured in animals through owner-completed questionnaires (objective list approach) (34). “An animal's quality of life can be classified as: a life not worth living, a life worth living and a good life.” “Assessment of an animal's quality of life should cover its welfare throughout its life, up to and including the manner of its death. On balance the positive experiences should still outweigh the negative over the animal's lifetime” (5)
A life worth living	“A life worth living is a statement about an animal's quality of life” […]. “A socially acceptable quality of life” (5) (Note that (29) challenges the concept as it is based on human judgement)	All vital needs, most mental needs and some wants are met. Good outweighs the poor welfare. To be assessed in accordance with the Five Freedoms (5)
A good life	The concept of “a good life” recognizes the distinction that an animal's quality of life is over and beyond that of a life worth living. Also defined as ‘welfare clearly beyond minimum [UK] legal standards' (5)	All vital needs, all mental needs and most wants are met. Good substantially outweighs the poor welfare. To be assessed in accordance with the Five Freedoms (5)

*Definitions and approaches were retrieved from a structured literature search based on the first use of the term or the most clearly described term, in order to provide a concise selection that illustrates the heterogeneous, and sometimes overlapping, terminology and concepts used to date. Underlined words or phrases gave rise to the two views: “hedonic positive welfare” and “positive welfare balance”*.

Many of the terms used to refer to positive (animal) welfare are similar to those used in (human) positive psychology for similar constructs (35). Positive psychology developed as a sub-field of human psychology that focuses on human thriving (35), and according to which human well-being does not just depend on treating pathology, weakness and damage but also on positive subjective experiences and positive individual qualities like strength and virtue (35). Positive psychology has been the subject of similar discussions and criticisms in its development [e.g., (36)] as we see today for positive welfare.

## Distinct Views on Positive Welfare

The scientific literature on positive welfare focuses on situations of positive valence as a common thread. Nonetheless, two main diverging views are prominent in the current literature. Some papers refer to positive welfare as (1) arising from likes and wants and their positive outcomes on welfare [e.g., (2)], whereas others allude to (2) an overall positive welfare state based on the effects of positive experiences outweighing the effects of negative experiences [e.g., (33)] (see Table 1 underlined text). To improve clarity, we propose that the first view could be coined “*hedonic positive welfare*” and the second view “*positive welfare balance*”. These two views differ in that they either consider only positive experiences, or the balance of positive and negative experiences; although both fully or partly focus on hedonic experiences and therefore the two views feed into each other. The field of positive psychology has debated similar aspects, defining its focus on positive emotions and positive qualities but acknowledging that (human) well-being ultimately involves a dialectical balance of positive and negative experiences (36). Positive welfare stands as a construct of its own when conceptualized as *hedonic positive welfare*, i.e., the effects of positive experiences on an animal's welfare. Considering positive welfare as *positive welfare balance*, i.e., encompassing both positive and negative experiences and their sum on the resulting (positive) welfare balance, overlaps with other concepts like quality of life, a good life, or happiness. Similarly, Lawrence et al. (30) identified four features from the positive welfare literature: *positive emotions* and *positive affective engagement* which pertains to the *hedonic positive welfare* view; and *quality of life* and *happiness* which pertains to the *positive welfare balance* view. The large majority of papers remain ambiguous or silent on their position or definition, involuntarily contributing to this conceptual uncertainty. Hence, we recommend that scientists clearly state their view when using the term, in order to prevent a dilution of the meaning of positive welfare. The view one chooses may also depend on the topic; for instance, receiving a treat fits the *hedonic positive welfare* view whereas free-range given the associated benefits and risks may fit better to the *positive welfare balance* view, and social play may be considered according to either view (see Framework section).

This discussion about positive welfare may also benefit from older discourses on the nature of human happiness and well-being. While a focus on the accumulation of positive experiences takes a more *hedonic* approach to positive well-being (38), Aristotle offered an alternative approach, termed *eudaimonia*, that presents a different perspective. In *Nicomachean Ethics* he writes, “For one swallow does not make a summer, nor does 1 day; and so too 1 day, or a short time, does not make a man blessed and happy” (39). This quote highlights the need to consider not only short-lasting emotions, but also longer-lasting states of contentment and life satisfaction that provide a more holistic view of positive well-being. Even those who contributed to early work parsing hedonic pleasure into “wanting” vs. “liking” acknowledge the concept of *eudaimonia* and argue for further studies to investigate how *eudaimonia* and *hedonia* relate to each other (40, 41). *Eudaimonia* questions the adequacy of simply accumulating positive experiences; for example, offering animals food treats may satisfy hedonic goals but a lifetime of consumption of tasty treats may lead to obesity that would violate eudaimonic goals (however, *hedonia* and *eudaimonia* are not necessarily always in conflict). Although *eudaimonia* does not appear to have found its way into the animal welfare science literature yet, it could become a third view. A hindrance may be the feasibility of its operationalization, given that the study of hedonic pleasure is more accessible with the current tools available (e.g., in behavioral biology) than the study of eudaimonic happiness, especially as approaches to *eudaimonia* in humans to date rely on self-report.

## The “Vienna Framework” for Structuring Positive Welfare Research

**Figure 1:**
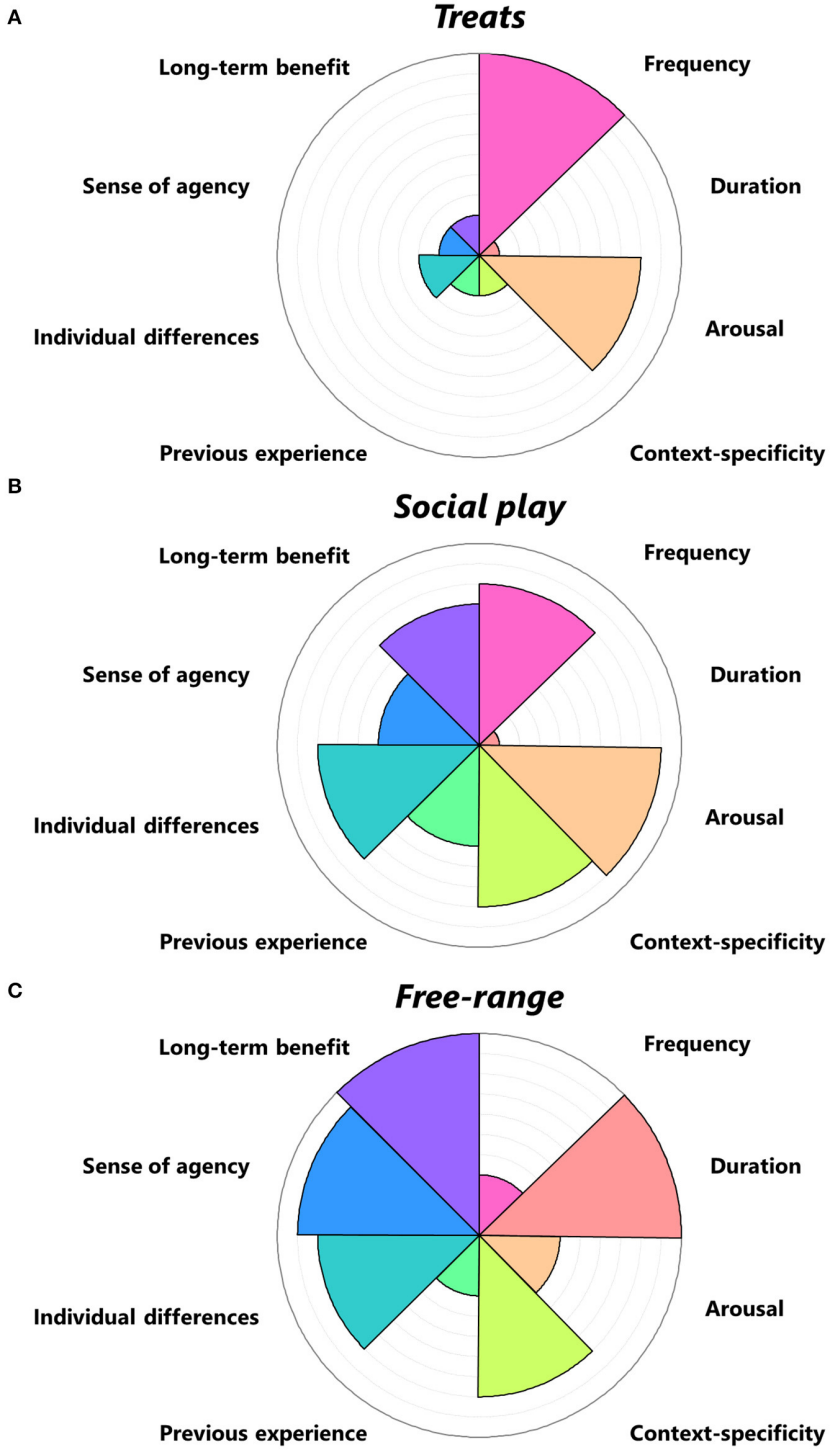
The “Vienna Framework.” The framework contains various parameters (facets) that are adjustable to represent a topic. Researchers are encouraged to utilize the discriminatory power of the facets by considering the importance of each facet to their research. Doing so may reveal tacit assumptions that may not be of primary interest but may nonetheless influence their research. Facets should be utilized as a heuristic tool by deciding the extent to which the facet matters, as conceptualized by a particular researcher. However, researchers are cautioned against using the surface area as a direct indicator of welfare output because facet values are not referenced to any validated metric, and because a high value means a facet matters more for the topic but does not necessarily mean greater welfare (e.g., for Context-specificity or Individual differences). Each of the three framework representations shows a different topic – **(A)** giving treats, **(B)** social play, and **(C)** free-range - to provide an example of how to employ the facets. For example, **Frequency** may matter more for the topic of Treats given the number of treats may vary widely; whereas **Duration** may matter less given the duration of eating each treat varies minimally. **Arousal** is typically high in Social play as it includes behaviors such as chasing, but typically low for Social huddling as it includes behaviors such as sleeping. **Context-specificity** may matter more for Social play and Free-range, as the resultant outcome on welfare depends on the social context in Social play and the broader environmental context in Free-range; in contrast, Treats probably result in a comparable welfare outcome regardless of the context. **Previous experience** may matter more for Social play if animals in the study have a rich history of group living, but may matter less if animals have a standardized or uniform social history; and may matter less for Treats and Free-range as these provisions are thought to improve welfare regardless of their history with them. **Individual differences** may matter more to Social play as animals may vary on sociability, ranging from shy to gregarious, and similarly for Free-range; but may matter less to Treats as animals tend to respond to these provisions more uniformly. A **Sense of agency** is encouraged in Free-range as the animal engages with its environment, discouraged in offering Treats as the animal passively receives Treats, or conditional in Social play as it partly depends upon the potential play partner. **Long-term benefit** should be utilized by determining whether the topic has the potential to provide adaptive advantages, as play behavior has been suggested as “training for the unexpected” (37), but the provision of Treats may not lead to adaptive advantages.

As discussed above, there is a plurality of terms and perspectives in the literature on positive welfare. To help lay out differences, we propose a framework that is applicable across the different views. The “Vienna Framework” is comprised of several facets of particular situations or behaviors for mapping one's own operational position on the positive welfare research landscape (Figure 1). The facets were derived from repeated discussions, based on knowledge of the existing literature, and centered around the question “What are important aspects for positive welfare?”. Although this framework is applied here to the topic of positive welfare, it is likely to be applicable to animal welfare more generally.

### Proposed Use of the Framework

The purpose of the “Vienna Framework” is to encourage researchers to consider the relevance of each of the facets of the framework for their own research in order to structure research on positive welfare. The framework helps to recognize and/or explicitly state the approach used to study positive welfare; for instance, according to the following aspects:

#### Focus

The framework can be used to structure one's research, either by thinking about how one's research topic relates to each facet (Figure 1 when focusing on the different facets within one topic), or by recognizing that one focuses on a specific facet(s) and does not consider other facets, e.g., one may be particularly interested in the role of individual differences on the animals' responses to stroking by a human. Moreover, differences between researchers studying the same topic, e.g., play behavior, may be illuminated, e.g., one researcher may focus on the frequency of play bouts while another researcher focuses on the duration of single play bouts.

#### Intervention

The framework can be used to show how interventions affect different facet(s), e.g., enriched animals may derive a greater sense of agency than non-enriched animals.

#### Topic

The framework can be used to compare different topics according to the same facet(s), e.g., play behavior may rate high on arousal whereas social affiliation may rate low (Figure 1 when comparing facets across treatments).

### Applying the Framework

The three proposed uses of the framework (1) Focus, (2) Intervention, and (3) Topic can be clarified by the following example sentence: A researcher may be studying *play behavior* (3) for its *adaptive significance on locomotor mobility* (1) *between different pigs provided with access to an alleyway or not* (2), whereas another researcher studies *social affiliation* (3) by the *duration* of contact (1) between animals *provided with familiar or unfamiliar partners* (2). This example illustrates how guidelines that help distinguish between different operational approaches can help clarify later discrepancies in interpretation.

This framework is neither exhaustive as other facets may be added with the continuing development of the field, nor exclusive as some facets may overlap in some instances. Some facets are easier to assess or better understood than others. Furthermore, knowledge may be too limited to evaluate all facets of positive welfare with the same degree of depth. Researchers are welcome to create customized visualizations of their own welfare topic by considering the importance of each facet to their conceptualization and assigning a value in the attached spreadsheet (see Supplemental Material).

We provide below examples for each listed facet.

### Frequency

Given that positive welfare is composed of elements that are inherently rewarding, animals usually aim to seek and repeat these experiences, thereby increasing their frequency and/or duration. The frequency may depend on the nature of the positive experience and the needs it fulfills, which is well-known from motivation tests (42). An increased frequency of particular behaviors is suggested to reflect positive welfare, e.g., in the case of allogrooming in primates (43), whereas a decreased frequency of, for example, brushing activity may be used as an early warning sign of morbidity in dairy cows (44).

### Duration

Positive welfare can last for various lengths of time, including short-term positive emotions and longer-term positive mood for example (45). It is therefore important to clarify whether the focus is on a potentially short-term positive response (e.g., when receiving a treat) or a potentially longer-lasting state (e.g., when being kept in an environment with varying enrichment). Habituation to positive stimuli may be a concern as the benefits of a specific positive experience can diminish over time; note that this is also true for Frequency. For example, the animal's interest in environmental enrichment materials (e.g., straw, hay, ropes, etc.) may diminish after a few days of exposure (46).

### Arousal

Positive welfare is often thought of in terms of high arousal activities such as play or anticipation, possibly due to their salience. Nevertheless, positive low arousal experiences have also been suggested like relaxation and sun-basking (47), or lying in body contact and other types of socio-affiliative interactions (48).

### Context-Specificity

What constitutes a positive situation may be contextual, meaning that something positive in one context may be less positive in another context or at another time. Lower contextual influences help ensure a more uniform benefit to positive welfare. For example, outdoor access can be highly valued by animals but varies according to many factors including the resources provided outside, time of the day and season of the year in meat chickens for instance (49). In the case of play, Held, and Ŝpinka (50) emphasize that play behavior is rewarding but “does not consistently reflect favorable environmental conditions”, hence dissociating the behavior from the context.

### Previous Experience and Current State of the Animal

Previous experience affects an animal's perception of a stimulus and possibly its expectations. For instance, gentle human-animal contacts can induce a positive perception of humans by the animal (51). Thus, positive situations may be a matter of relative difference, based on a positive discrepancy between the actual situation and the animal's expectation, or linked to the novelty of the situation.

It can also depend on the animal's current welfare state by modifying reward sensitivity such that negative states may render the animal more sensitive to positive experiences (52) or conversely induce anhedonia (53, 54). Caution is warranted that the alleviation of suffering does not mean positive welfare. Rather, positive welfare arises from situations and the resulting experiences that the animal would voluntarily seek again.

### Individual Differences

Animal personality (55), including coping styles (56) and individual differences more generally (57) may modulate how an animal perceives a presumably positive experience and/or the effect of this experience on the animal's welfare. For instance, individual laying hens vary greatly in their motivation to work for access to substrates for dust bathing (58), and there are substantial within- and between-litter differences in play behavior in piglets (59). Individual differences may be greater for positive experiences than for negative experiences, given that selection pressure is presumably greater for adaptive responses to threatening situations, whereas positive experiences have been suggested as opportunities taken when the environment is safe [e.g., (31, 60)]. However, this hypothesis is yet to be tested.

### Sense of Agency

The animal as being a central actor of its life is frequently cited in the positive welfare literature, with positive welfare being encouraged by a sense of control (6), realizing goals (2), agency (61), or control effectiveness (62). Whereas, control has a long tradition as a determinant of animal welfare (63), agency and control effectiveness have found their way into animal welfare science more recently (37, 61, 62). Ŝpinka (61) defines agency as an “inner-motivated behavioral engagement with the environment”. Agency has been proposed to comprise various levels such as action-driven agency (behaving actively to satisfy current needs) and competence-building agency (enabling the animals to gather knowledge and enhance their skills) (61). For example, some positive welfare situations facilitate a sense of agency (e.g., provision of enrichment items), some discourage it (e.g., receiving food treats), and some could conditionally facilitate or discourage it (e.g., provision of social partners). The development of operational methodologies is needed to investigate the extent to which positive welfare builds up these strengths and skills, and its potential link to *eudaimonia*.

### Long-Term Benefit

Positive welfare may safeguard welfare through enhanced stress resilience (64, 65) or allostasis (66) with greater competency or flexibility (37, 60, 67) that becomes advantageous during or after challenges. For instance, play behavior is postulated to enhance flexibility to cope with unexpected situations (37). These protective (preventive) or counteracting (therapeutic) effects go beyond what is achieved by the mere absence of suffering. The potential benefits could be classified in the physical, psychological, social, and/or health domains. Studies often report smaller effects on welfare outcomes for positive as compared to negative experiences (4, 51, 60). Nevertheless, in humans, satisfaction with one's life is determined to a greater extent by positive experiences than by the adversity that they faced (68), and these positive aspects in turn determine longevity, health and well-being in the longer-term (69).

## Conclusions

Positive welfare opens up opportunities through which animal welfare science can identify what should be provided to animals rather than what should be avoided. Although the literature proved heterogeneous, we identified two distinct views that we coined “*hedonic positive welfare*” and “*positive welfare balance*,” with *eudaimonia* possibly emerging as a third view. Complementarily, we propose a framework intended to structure the research on positive welfare through the empirical study of different facets of positive welfare. We encourage researchers to explicitly report their conceptual view and operational approach (e.g., using the framework, see Supplementary Material) to clarify the field of positive welfare.

## Author Contributions

J-LR, JY, IC, and SH reviewed and interpreted the literature and wrote the draft of the manuscript. J-LR initiated the idea. SH elaborated on the framework. IC drafted Table 1. JY drafted Figure 1. All authors contributed to the article and approved the submitted version.

## Conflict of Interest

The authors declare that the research was conducted in the absence of any commercial or financial relationships that could be construed as a potential conflict of interest.
